# Two homolog wheat Glycogen Synthase Kinase 3/SHAGGY - like kinases are involved in brassinosteroid signaling

**DOI:** 10.1186/s12870-015-0617-z

**Published:** 2015-10-13

**Authors:** Thomas Bittner, Sabine Nadler, Eija Schulze, Christiane Fischer-Iglesias

**Affiliations:** Cell Biology, Faculty of Biology, Albert-Ludwigs-University Freiburg, Schaenzlestr. 1, 79104 Freiburg, Germany

**Keywords:** GSK-3/SHAGGY-like kinases, *Triticum aestivum*, Brassinosteroids, Embryonic patterning, Seedling growth response

## Abstract

**Background:**

Glycogen Synthase Kinase 3/SHAGGY-like kinases (GSKs) are multifunctional non-receptor ser/thr kinases. Plant GSKs are involved in hormonal signaling networks and are required for growth, development, light as well as stress responses. So far, most studies have been carried out on *Arabidopsis* or on other eudicotyledon GSKs. Here, we evaluated the role of TaSK1 and TaSK2, two homolog wheat (*Triticum aestivum*) GSKs, in brassinosteroid signaling. We explored in addition the physiological effects of brassinosteroids on wheat growth and development.

**Results:**

A *bin2-1* like gain-of-function mutation has been inserted respectively in one of the homoeologous gene copies of *TaSK1* (*TaSK1-A.2-1)* and in one of the homoeologous gene copies of *TaSK2 (TaSK2-A.2-1). Arabidopsis* plants were transformed with these mutated gene copies. Severe dwarf phenotypes were obtained closely resembling those of *Arabidopsis bin2-1* lines and *Arabidopsis* BR-deficient or BR-signaling mutants. Expression of BR downstream genes, *SAUR-AC1*, *CPD* and *BAS1* was deregulated in *TaSK1.2-1* and *TaSK2.2-1* transgenic lines. Severe dwarf lines were partially rescued by Bikinin beforehand shown to inhibit TaSK kinase activity. This rescue was accompanied with changes in BR downstream gene expression levels. Wheat embryos and seedlings were treated with compounds interfering with BR signaling or modifying BR levels to gain insight into the role of brassinosteroids in wheat development. Embryonic axis and scutellum differentiation were impaired, and seedling growth responses were affected when embryos were treated with Epibrassinolides, Propiconazole, and Bikinin.

**Conclusions:**

In view of our findings, TaSKs are proposed to be involved in BR signaling and to be orthologous of *Arabidopsis* Clade II GSK3/SHAGGY-like kinases. Observed effects of Epibrassinolide, Propiconazole and Bikinin treatments on wheat embryos and seedlings indicate a role for BR signaling in embryonic patterning and seedling growth.

**Electronic supplementary material:**

The online version of this article (doi:10.1186/s12870-015-0617-z) contains supplementary material, which is available to authorized users.

## Background

Glycogen Synthase Kinase 3 (GSK3)/SHAGGY (SGG)-like-kinases regulate a broad range of fundamental biological processes in eukaryotes. Land plant GSKs are involved in flower, embryonic, stomata development, as well as in light and stress responses [1–5]. Plant GSKs in contrast to animal GSKs are encoded by a multigene family and have been grouped into four major clades [6–8].

Five out of 10 Arabidopsis GSKs (ASKs) are key signaling players in the brassinosteroid (BR) pathway. These ASKs are the three clade II ASKs, ASKiota, ASKdzeta and ASKeta/BIN2 (BRASSINOSTEROID INSENSITIVE 2), the clade III ASKtheta and the clade I ASKgamma [9–15].

These kinases act as negative regulators of BR signaling pathway [10]. BIN2 phosphorylates BR response transcription factors BRI1-EMS-SUPPRESSOR1 (BES1) and BRASSINAZOLE-RESISTANT1 (BZR1) to influence their subcellular localization [16, 17], to target BZR1 for protein degradation [18], and to impact both binding to target promoters and transcriptional activity of BES1 [12]. BES1 and BZR1 regulate the expression of numerous BR target genes leading to a whole range of BR physiological responses [19]. They influence also the expression of genes involved in other signaling pathways such as auxin and GA signaling [19].

BIN2 kinase activity and protein level are negatively regulated by BR signaling through respectively dephosphorylation of a conserved tyrosine residue and proteasome mediated protein degradation [13, 20]. The study on BIN2 kinase inactivation has closed the gap in the understanding of BR signaling from the perception of BR to the inactivation of BIN2. Indeed, upon its phosphorylation by the BR receptor kinase BRI1 (BRASSINOSTEROID INSENSITIVE 1), BSK1 (BR-SIGNALING KINASE 1) is released from the receptor complex and interacts with BSU1 (BRI SUPPRESSOR 1) phosphatase [13]. Binding of activated BSU1 to BIN2 is proposed to lead to the dephosphorylation of the conserved Tyr 200 and inactivation of BIN2 [13].

Two homolog wheat (*Triticum aestivum*) GSKs called TaSK1/TaSK2, having 88 % identity at the protein level were identified and characterized in a previous study [8]. Sequence alignment and chromosome localization using nullisomic-tetrasomic lines substantiate three expressed gene copies *TaSK1-A,B,C* and *TaSK2-A,B,C* located on homoeolog chromosomes i.e., related chromosoms deriving from different genomes of allopolyploid species. Identity at the protein level among the three TaSK1 homoeologs and among the three TaSK2 homoeologs was in both cases higher than 98 % [8]. Analysis of predicted protein sequence pointed out a clear GSK3/SGG signature for TaSK1 and TaSK2 [8]. *In vitro* kinase assays showed that both were functionally active kinases [8]. Phylogenetic analysis of land plant GSKs indicated that TaSK1 and TaSK2 belong to clade II of plant GSKs, the *Arabidopsis* members of which are all involved in brassinosteroid signaling [8].

The present study addresses the question whether TaSKs being members of clade II are involved in BR signaling as reported for *Arabidopsis* group II ASKs.

For this purpose, a *bin2-1* like gain-of-function mutation has been inserted in *TaSK1-A* and in *TaSK2-A. Arabidopsis* plants were then transformed with the mutated gene copies. The phenotypes of the transgenic lines have been investigated and BR target gene expression levels have been analyzed in representative transgenic lines. Rescue experiments of the severe phenotypes were conducted by means of the ASK chemical inhibitor Bikinin, beforehand shown to inhibit TaSKs.

Information on the role of BR signaling in Liliopsida resp. *Poaceae* development in particular wheat development is so far limited. Therefore, the impact of compounds interfering with BR signaling or changing BR levels has been evaluated on different wheat developmental stages.

## Results

### *TaSK1.2-1 and TaSK2.2-1* mutated gene copies expressed in *Arabidopsis* led to phenotypes reminiscent of BR-signaling mutant phenotypes

Previous phylogenetic analyses pointed out that TaSK1-A,B,C and TaSK2-A,B,C are members of plant GSK clade II [8]. All three *Arabidopsis* members of clade II, ASKiota, ASKdzeta and BIN2, are involved in Brassinosteroid (BR) signaling [9–12]. The catalytic domains of TaSKs share 90–91 % identity with the catalytic domain of BIN2 [8]. *Arabidopsis* lines expressing *TaSKs* were generated to address the question whether these clade II wheat members have a function in BR signaling. TaSKs contain the highly conserved TREE motif within their catalytic domain [8]. *Bin2-1* gain-of-function mutation localizes to this motif [9–11]. The mutated BIN2-1 protein is more stable than the wild type protein and is not depleted by BL treatment as the wild type form [20]. The *bin2-1* mutation blocks the dephosphorylation of BIN2 residue Tyr 200 by BSU1 causing BR insensitivity [13]. The phenotype of these mutants is resembling the phenotype of BR-deficient or BR-signaling mutants [9–11]. A *bin2-1* like mutation (E263K) has been inserted in *GFP:TaSK1-A* and *GFP:TaSK2-A* transgenes via site directed mutagenesis. *Arabidopsis thaliana* ecotype Columbia plants were transformed with these constructs.

**Fig. 1:**
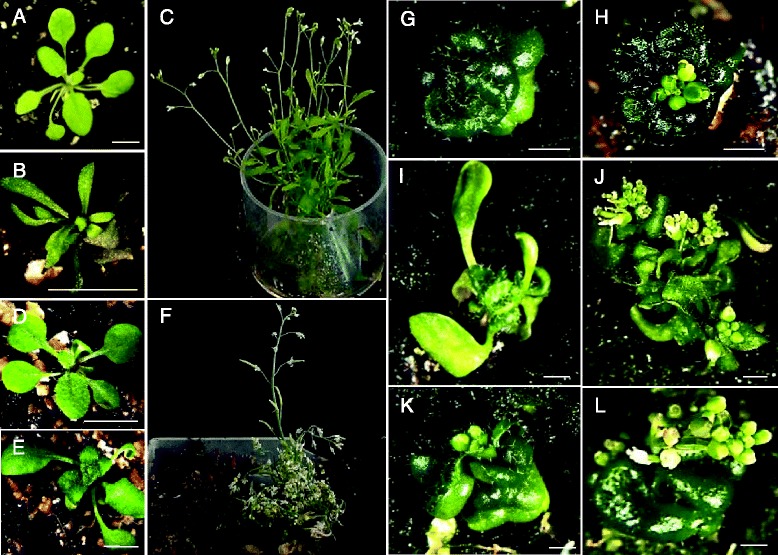
Phenotypes of *TaSK1.2-1 and TaSK2.2-1 Arabidopsis* transgenic lines. **a**, **d** 26 and 22 days old wild type Columbia plants. **b**, **c** 28 and 66 days old *TaSK1.2-1.P4T23* line. The 28 days old plant had leaves with thin blades. After 66 days, the plant with a reduced apical dominance had reached a size of 11 cm while wild type plants had a size of at least 40 cm. **e**, **f** 22 and 57 days old *TaSK1.2-1.P6T26* line. After 57 days, the plant reached a size of 6 cm whereas wild type plants had a size of 40 cm. **g** 43 days old *TaSK1.2-1.P3T4* severe dwarf line. **h** 43 days old *TaSK2.2-1.P2T2* severe dwarf developing flowers. **i**, **j** 43 and 60 days old *TaSK1.2-1.P3T1* dwarf line showing leaves with thin blades, and later flowers. **k**, **l** 43 and 60 days old *TaSK1.2-1.P3T6* severe dwarf differentiating flowers. Bars in (**a**), (**b**), (**d**), (**e**) represent 5 mm while bars in (**g**) to (**l**) represent 1 mm

Besides plants having a normal size and an apparent wild type phenotype, a range of significantly shorter plants were observed in the T1 generation. Observed phenotypes were including shorter stems, reduced apical dominance, elongated thinner blade leaves, delayed flowering, shorter siliques and reduced fertility (Fig. 1b, c, e, f). In addition, severe dwarf phenotypes having compact dark green and downwards rolled thicker leaves were consistently observed, some of them developing flowers (Fig. 1g, -l). These flowers were in direct contact with the compact thicker leaves due to extremely short internodes. Few transgenic lines showed in addition an altered flower patterning (data not shown).

**Fig. 2:**
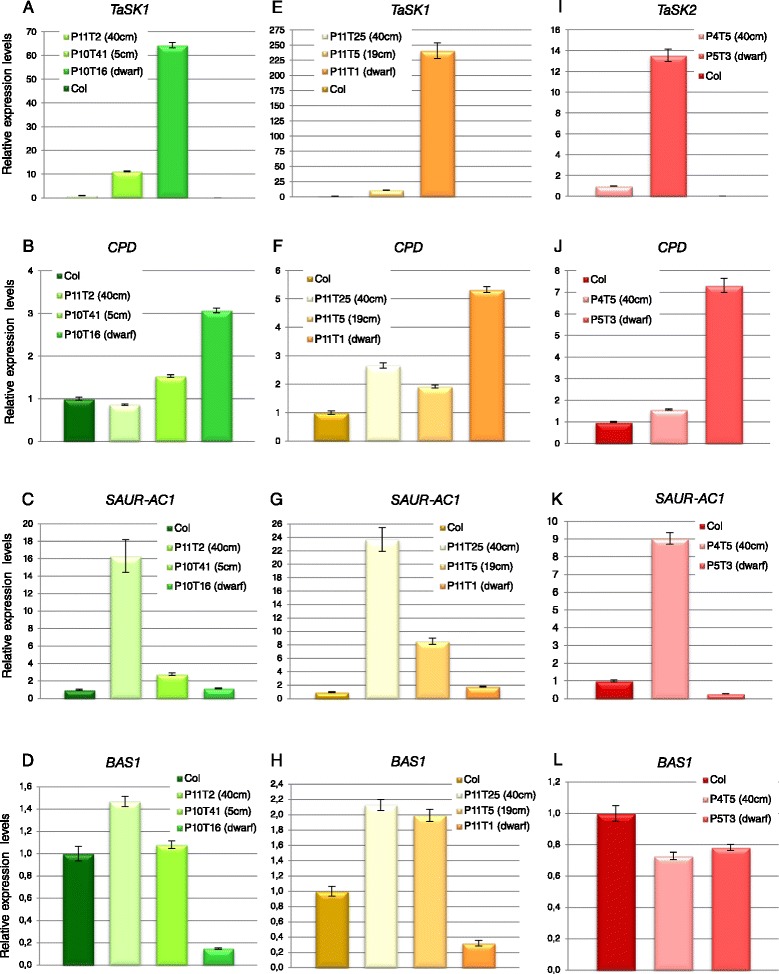
BR target gene expression levels in *TaSKs.2-1* transgenic lines. mRNA level of BR-biosynthetic *CPD*, BR-upregulated *SAUR-AC1* and BR inactivation *BAS1* genes were quantified by qRT-PCR in *TaSK1.2-1* (**a**-**h**) and in *TaSK2.2-1* (**i**-**l**) lines. Relative expression levels in representative lines having same size and morphology as wild type plants (approx. 40 cm), in representative semi-dwarfs and in representative severe dwarfs (with and without flowers, referred as dwarf) were compared to expression levels in Columbia plants. In the recorded samples, 36 out of 180 *TaSK1.2-1* transgenic plants were severe dwarfs and 69 had a normal size. Eleven out of 42 *TaSK2.2-1* transgenic plants were severe dwarfs and 18 had a normal size. All results are means +/- standard deviations. Individual reactions were done in triplicate (technical replicates). Expression levels were normalized to those of *Polyubiquitin10*. Similar results were obtained when normalizing expression levels to those of *EF-1alpha* (Additional file 1)

The severity of the phenotype correlated with the levels of *TaSK1 and 2* transcripts (Fig. 2a, e, i; Additional file 1, A). Severe dwarfs expressed the highest *TaSK1 and 2* levels while the lowest levels were measured in transgenic plants having the same size and morphology as wild type plants (Fig. 2a, e, i; Additional file 1, A). Semi-dwarf lines showed intermediate *TaSK1* levels (Fig. 2a, e; Additional file 1, A).

In summary, the observed phenotypes were reminiscent of the *bin2-1* mutant phenotypes observed in *Arabidopsis* [9–11].

### BR target gene expression was deregulated in *TaSKs.2-1* transgenic lines

RNA levels of BR-induced *SAUR-AC1,* BR inactivation *BAS1* and BR biosynthetic *CPD* genes were quantified by real-time PCR to verify whether BR downstream target gene expressions were deregulated in *TaSKs.2-1* transgenic lines. *SAUR-AC1* and *CPD* have been selected respectively as BES1 and BZR1 target genes, BES1 and BZR1 being negatively regulated by BIN2 kinase [19, 21–23]. Data documented in the literature indicate that *SAUR-AC1* and *BAS1* are up-regulated while *CPD* is down-regulated by application of exogenous BL on wild type *Arabidopsis* seedlings [14, 24–26]. Furthermore *bin2-3* loss-of-function mutants show an increase of *SAUR-AC1* and a decrease of *CPD* transcript levels compared to wild type plants [14].

As already mentioned, a correlation was observed between the severity of the phenotype of *TaSKs.2-1* lines and expression levels of *TaSKs* (Fig. 2a, e, i; Additional file 1, A). This suggests that higher TaSKs.2-1 protein levels were associated with a stronger inhibition of BR signaling.

As shown in Fig. 2, down-regulation of *CPD* and up-regulation of *SAUR-AC1* and *BAS1* by BR signaling were the strongest impaired in the lines expressing the highest levels of *TaSK1* and *TaSK2,* and which were the most compromised morphologically. Indeed, *CPD* transcript levels were higher in severe dwarfs than in morphological normal transgenic plants (Fig. 2b, f, j; Additional file 1, B). Furthermore, transcripts levels of *SAUR-AC1* and *BAS1* were lower in severe dwarfs compared to the other transgenic lines (Fig. 2c, g, k and d, h; Additional file 1, C). The increase of *CPD* and the decrease of *SAUR-AC1* and *BAS1* levels in transgenic lines were proportional to the severity of the phenotype (Fig. 2; Additional file 1). *BAS1* expression levels in the tested severe dwarf and morphologically normal *TaSK2* lines were not significantly different to one another and to Columbia control plants (Fig. 2, l). Although deeper investigation is required, this observation may reflect functional differences between the two wheat homologs in the regulation of this downstream target gene.

In conclusion, expressions of *SAUR-AC1*, *CPD* and *BAS1* were deregulated in *TaSKs.2-1* lines. Expression patterns of these BR downstream genes were in accordance with the effect of a negative regulator of BR signaling.

### Bikinin inhibited TaSK kinase activity

The activity of 7 ASKs including all clade II *Arabidopsis* members is inhibited by Bikinin [(4-[(5-bromo-2-pyridinyl)amino]-4-oxobutanoic acid; 27]. Bikinin activates BR signaling in *Arabidopsis* by directly binding to BIN2 and acting as ATP competitor [27]. Predicted TaSK1 and TaSK2 protein sequences include the motif **M**E**YV** reported to contain key residues for docking of Bikinin [8, 27].

In a previous study, we showed by means of *in vitro* kinase activity assays that TaSK1 and TaSK2 were functionally active kinases [8]. In the present study, *in vitro* kinase assays were performed in the presence and absence of Bikinin to evaluate whether this compound was inhibiting the two wheat members of clade II.

Besides TaSK1 and TaSK2 (longest ORF), Arabidopsis BIN2, and wheat TaGSK1 were overexpressed in *E. coli* as GST fusion proteins and affinity purified in native conditions. TaGSK1 is involved in salt tolerance and is one of the few other wheat GSK-like-kinases whose function has been so far investigated [28]. Phylogenetic analysis showed that TaGSK1 is a member of GSK clade I [8].

**Fig. 3:**
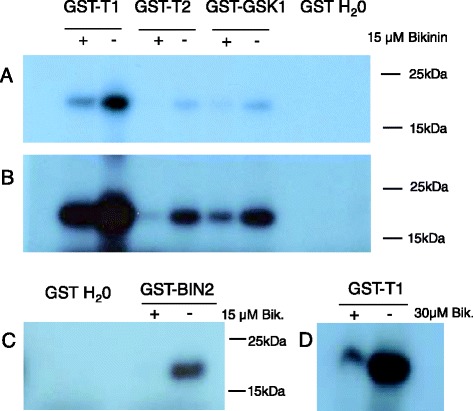
*In vitro* kinase activity of TaSKs in the presence of Bikinin. Phosphorylation activity of purified TaSK1, TaSK2, TaGSK1 and BIN2 on a myelin basic protein fragment (18.454 kDa) was determined by *in vitro* kinase assays using ATP γP^32^. Kinase activity was analyzed in the presence and absence of Bikinin added at a concentration of 15 or 30 μM. After kinase reaction, samples were loaded on a 12 % SDS PAGE gel. After migration, the gel was directly exposed to an X-ray film for either 80 min at room temperature (**a**), o/n at room temperature (**b**), o/n at -80 °C (**c**), o/n at room temperature (**d**)

An almost total inhibition of BIN2 activity with less than 5 % residual activity was observed in the presence of 15 μM Bikinin (Fig. 3c) as reported previously in the literature [27]. Bikinin added at a concentration of 15 μM was clearly inhibiting the kinase activities of TaSK1, TaSK2 and TaGSK1 (Fig. 3a and b). In our experimental conditions, inhibition was ranging from very strong for TaSK2 with approx. 5 % residual activity to strong for TaSK1 and TaGSK1 with a residual activity in the range of 30 % (Fig. 3a and b). Increasing the concentration of Bikinin to 30 μM was leading to a stronger inhibition of TaSK1 with significantly less than 30 % residual activity (Fig. 3d).

In conclusion, Bikinin was inhibiting not only eudicotyledon- but also Liliopsida-GSKs as shown for three wheat GSKs belonging to clade II and I.

### *TaSK1.2-1* and *TaSK2.2-1* severe dwarfs were rescued by Bikinin

**Fig. 4:**
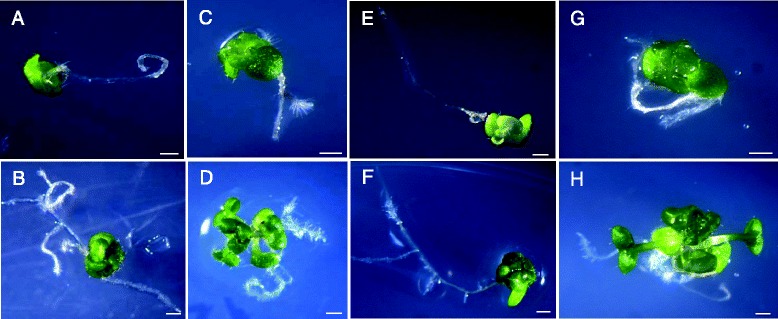
*TaSKs.2-1* severe dwarf phenotypes rescued by Bikinin. **a**,**b**,**c**,**d** depict *TaSK1.2-1* lines. *T1-P7SevereDwarf2* at day 0 (**a**) and day 7 (**b**) on 0.3 % DMSO control medium. *T1-P7SevereDwarf1* at day 0 (**c**) and day 7 (**d**) on medium containing 30 μM Bikinin. E,F,G,H depict *TaSK2.2-1* lines. *T2-P7SevereDwarf4* at day 0 (**e**) and day 7 (**f**) on 0.3 % DMSO control medium. *T2-P7SevereDwarf1* at day 0 (**g**) and day 7 (**h**) on medium containing 30 μM Bikinin. Bars represent 1mm

*TaSK1-A.2-1* and *TaSK2-A.2-1* severe dwarf *Arabidopsis* lines were transferred to a Bikinin supplemented medium (Fig. 4). After 7 days of Bikinin treatment, they showed a significant increase in hypocotyl length, longer bending petioles, more blade-shaped and pale green leaves (Fig. 4d and h). This observation indicates that Bikinin was able to rescue partially severe dwarf *TaSK.2-1* phenotypes.

**Fig. 5:**
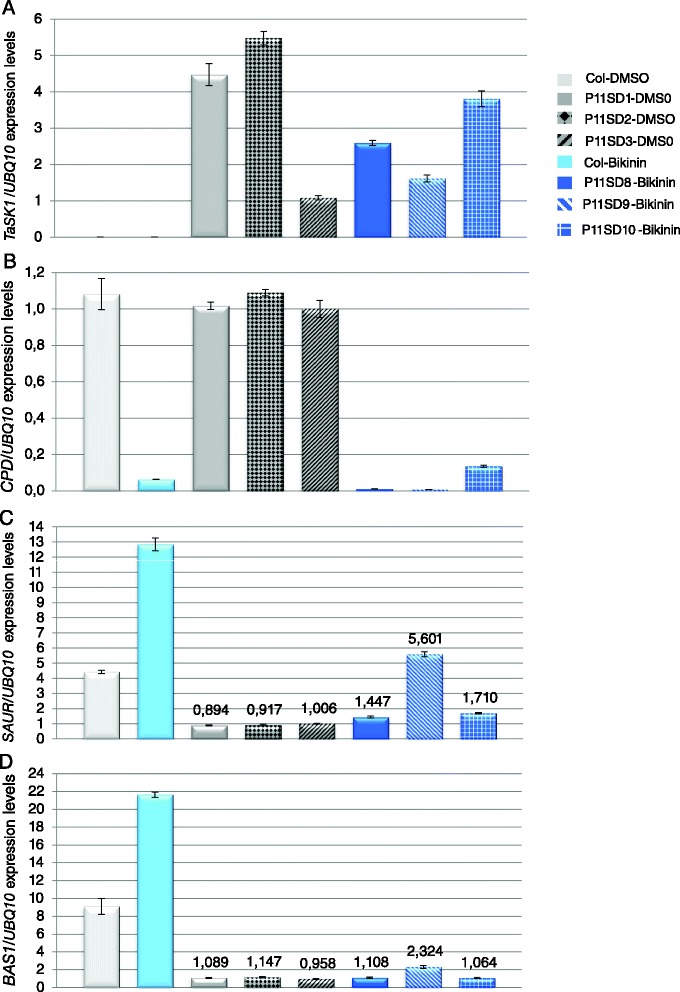
Effect of Bikinin on BR target gene expression levels in *TaSK1.2-1* severe dwarf lines. mRNA levels of *TaSK1* (**a**), BR-biosynthetic *CPD* (**b**), BR-upregulated *SAUR-AC1* (**c**) and BR inactivation *BAS1* (**d**) genes were quantified by qRT-PCR in *TaSK1-A.2-1* severe dwarf (SD) lines either treated with 30 μM Bikinin or grown on control medium for 8 days. mRNA levels of BR target genes were in parallel quantified in Columbia seedlings either treated with 30 μM Bikinin or grown on 0.3 % DMSO for 7 days. All results are means +/- standard deviations. Individual reactions were done in triplicate (technical replicates). Expression levels were normalized to those of *Polyubiquitin10*. Similar results were obtained when normalizing expression levels to those of *EF-1alpha* (Additional file 2)

Transcript levels of BR-inducible *SAUR-AC1*, BR biosynthetic *CPD* and BR inactivation *BAS1* genes were quantified by qRT- PCR to determine whether Bikinin treatment affected BR target gene transcription of *TaSK1.2-1* severe dwarfs (Fig. 5, Additional file 2). A strong decrease of *CPD* as well as a strong increase of *SAUR-AC1* and *BAS1* levels were recorded in Columbia wild type seedlings subjected to 30 μM Bikinin treatment (Fig. 5b-d, Additional file 2, B-D) as reported in the literature [27]. A strong decrease of *CPD* expression was observed in all severe dwarfs treated with Bikinin (Fig. 5b; Additional file 2, B). Up-regulation of *SAUR-AC1* transcription was observed in severe dwarfs treated with Bikinin (Fig. 5c; Additional file 2, C). A significant increase of *BAS1* transcript levels was recorded for line P11SD9, however levels in the same range as those of control lines were obtained for lines P11SD8 and P11SD10 (Fig. 5d; Additional file 2, D).

In conclusion, Bikinin treatment of *TaSK1.2.1* dwarf lines induced a change in the expression pattern of BR target genes. The expression pattern in Bikinin-rescued *TaSK1.2-1* lines was opposite to the one observed previously in *TaSK1.2-1* and *TaSK2.2-1* severe dwarf lines (Fig. 5/Additional file 2 and Fig. 2/Additional file 1 respectively). Furthermore, observed changes were resembling or were evolving towards expression patterns observed for *Arabidopsis* wild type plants treated with Bikinin [27].

### *TaSKs* were ubiquitously expressed in wheat tissues

*TaSK1 and 2* expression pattern in different wheat tissues was investigated as a first step towards exploring their role and the role of BR signaling in *Poaceae* development.

**Fig. 6:**
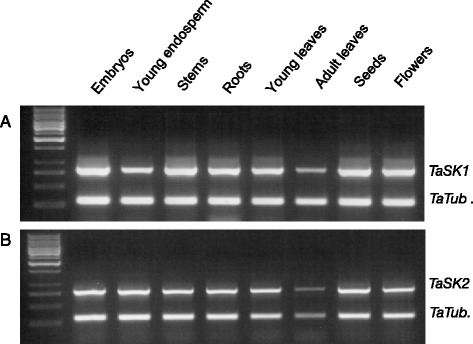
*TaSK1* and *TaSK2* expression levels in different wheat tissues. *TaSK1* (**a**) and *TaSK2* (**b**) expression levels in different wheat tissues were analyzed by means of semi-quantitative RT-PCR. Wheat *TUBULIN* was used as reference

*TaSK1* and *TaSK2* expression pattern were analyzed by semi-quantitative RT-PCR in embryos, young endosperm, stems, roots, young and adult leaves, seeds, flowers (Fig. 6). Both genes were expressed in all tested tissues at approximately the same levels except in adult leaves where *TaSK1* may be expressed at slightly lower levels (Fig. 6).

### BL, Bikinin and Propiconazole impacted the growth of wheat seedlings

**Fig. 7:**
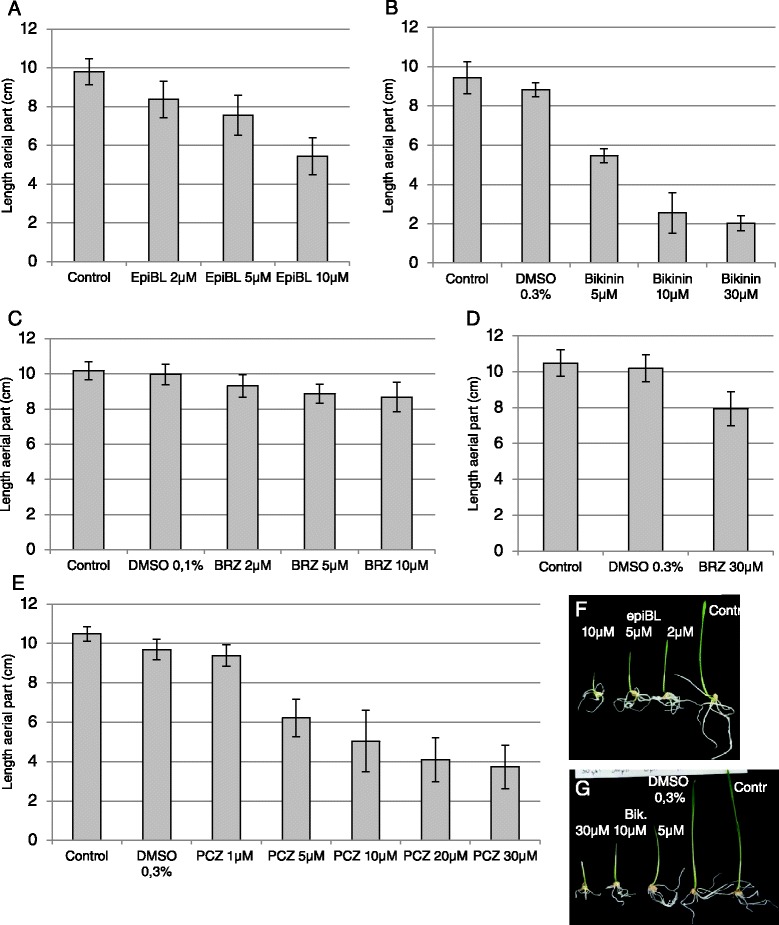
Response of *in vitro* grown wheat seedlings to epiBL and inhibitor treatments. Wheat seeds were sown and cultured for 6 days under white light (16h light/8h dark cycles) on ½ MS medium containing 1 % sucrose supplemented with different concentrations of epiBL (**a**, **f**), Bikinin (**b**, **g**), BRZ (**c**, **d**) and PCZ (**e**). Values represent mean values and standard deviations of seedling aerial part length ( *n* >15) measured after 6 days of culture

BRs promote hypocotyl elongation and inhibit root growth while brassinazole (BRZ), a BR biosynthesis inhibitor, reduces hypocotyl and root length of light grown *Arabidopsis* seedlings [29]. A significant increase in hypocotyl length is observed when light grown *Arabidopsis* seedlings are treated with Bikinin [27]. However, BR response is reported to be species-dependent [29]. Information about the effects of Epibrassinolide, compounds impairing BR synthesis and compounds activating BR signaling on wheat development is so far scarce. Consequently, we tested the growth response of wheat seeds sown and cultured *in vitro* for 6 days on medium supplemented with different concentrations of either Epibrassinolide (epiBL), Brassinazole (BRZ) or Bikinin (Fig. 7). Exogenously applied epiBL inhibited the growth of the aerial part of the seedling in a dose dependent manner (Fig. 7a; Additional file 3). The GSK inhibitor Bikinin was even more effective in the inhibition of seedling upper part growth and acted also in a dose dependent manner (Fig. 7b). Root growth was inhibited by both, epiBL and Bikinin, particularly at higher concentrations (Fig. 7f and g). By contrast, inhibition of aerial part growth observed upon BRZ treatment was much less effective even at high concentration (30 μM) (Fig. 7c and d). Monocots are apparently less responsive to BRZ [30]. Propiconazole (PCZ) is reported as a specific and an effective BR biosynthesis inhibitor [31]. In particular, maize seedlings have impaired mesocotyl, coleoptile and true leaf elongation upon application of PCZ [31]. Therefore PCZ was used in the present study as an alternative to BRZ. The compound was far more efficient as BRZ to inhibit the growth of the aerial part of the wheat seedling, the observed effect being dose dependent (Fig. 7e).

**Fig. 8:**
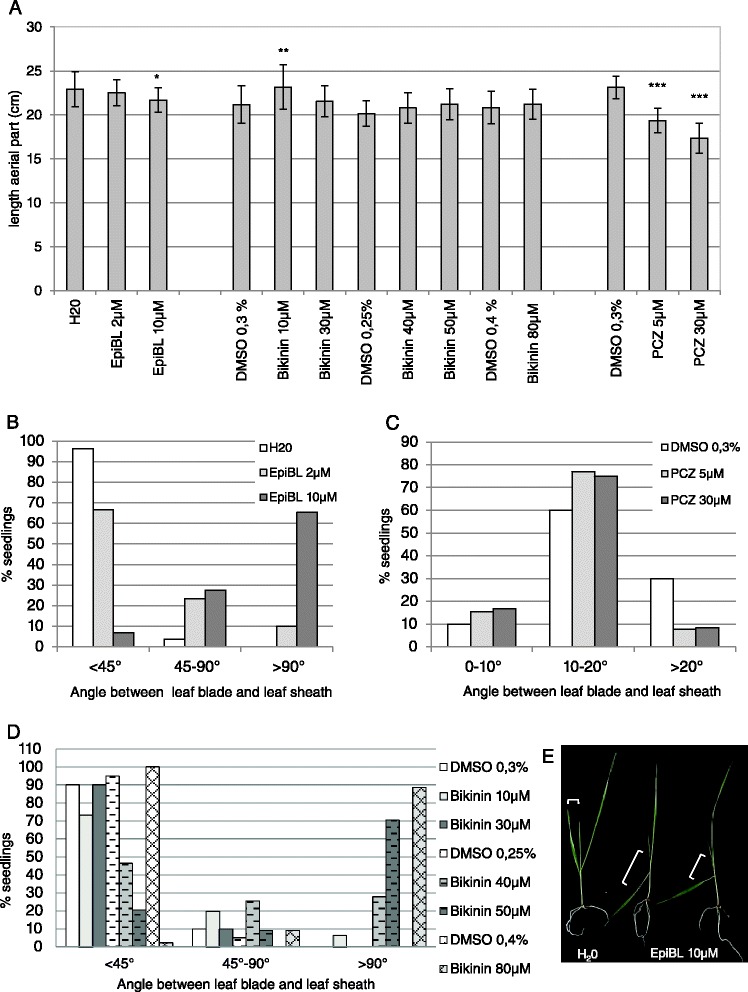
Response of wheat seedlings to epiBL, Bikinin and PCZ foliar spray. Selected concentrations of epiBL, Bikinin and PCZ were sprayed 4 times on the leaves of wheat seedlings grown for 14 days on vermiculite in an incubator under the same conditions as the *in vitro* grown seedling. Values in (**a**) represent mean values and standard deviations of seedling aerial part length measured after 14 days of culture. Values in (**b**), (**c**) and (**d**) represent the percentage of seedlings that had a given inclination angle (angle between the first leaf blade and sheath) upon epiBL (**b**), PCZ (**c**) and Bikinin (**d**) treatment after 14 days of culture. **e** shows representative inclination angles upon epiBL treatment. In independent experiments, 27–30 seedlings were monitored for each epiBL concentration and for the control. Thirty, 15, 30, 40, 43, 44, 41, 44 seedlings were recorded for respectively 0.3% DMSO, 10 and 30 μM Bikinin, 0.25 % DMSO, 40 and 50 μM Bikinin, 0.4 % DMSO, 80 μM Bikinin. Finally, 12–13 seedlings were measured for the control and each PCZ concentration. * T.test *p* <0.05 (*p* = 0.01026141 for 10 μM epiBL), ** T.test *p* <0.01 (*p* = 0.00787367 for 10 μM Bikinin).***T.test p <0.001 (*p* = 3.5079E-07 for 5 μM PCZ, *p* = 3.75455E-09 for 30 μM PCZ)

These results contrast with the reported effects of these compounds on *Arabidopsis* growth. BR growth responses depend on many factors such as plant cultivar, plant organ tested, concentration used and the method of BR application [32]. Thus, application of BR through the roots for few days is reported to decrease the growth of wheat seedlings while foliar application is stimulating the growth of the upper part of the seedlings [32]. To test whether the method of application influences the growth response, epiBL, Bikinin, and PCZ were sprayed at selected concentrations four times on the aerial part of seedlings (Fig. 8). No noteworthy change of plant size was observed at the epiBL and Bikinin concentrations tested (Fig. 8a). A slight increase of plant height may occur at 10 μM Bikinin (T.test *p* <0.01) whereas a small decrease may arise at 10 μM epiBL (T.test p <0.05) (Fig. 8a). Propiconazole (PCZ) treatment led as expected to a significant size reduction of the seedling aerial part (*p* <0.001) (Fig. 8a). However, a clear increase of the first leaf bending was observed with rising epiBL and Bikinin concentrations (Fig. 8b, d and e). This physiological response called lamina joint inclination that is the bending between the leaf blade and the leaf sheath is described as one of the most sensitive BR response in rice [33–35]. The first leaf of non-treated wheat seedlings was bending with an angle smaller than 45° while the first leaf of most seedlings treated with 10 μM epiBL bent with an angle greater over 90° (Fig. 8b and e). Bikinin impacted the inclination of the first leaf at concentrations over 30 μM (Fig. 8d). In particular, upon 50 and 80 μM Bikinin treatment, 70 % and 89 % of the seedlings respectively had an inclination angle over 90° (Fig. 8d). A slightly higher percentage of seedlings had a smaller angle between the first leaf sheath and leaf blade in the presence of PCZ compared to control conditions (Fig. 8c). Indeed, 77 and 75 % of the seedlings sprayed with 5 and 30 μM PCZ respectively had an inclination angle of 10 to 20° while only 60 % of the control seedlings had such a small angle (Fig. 8c). Thirty percent of the control seedlings showed an angle over 20° while about 8 % of seedlings sprayed with PCZ had such an angle (Fig. 8e). The effect of PCZ on the lamina joint inclination was independent of the concentration tested (Fig. 8c).

EpiBL, Bikinin, and PCZ were also sprayed at selected concentrations three times on the aerial part of plants grown in soil under greenhouse conditions (Additional file 4). The plants at the moment of the first foliar spraying were older than the seedlings tested in the previous experiment (15–22 days towards 6 days after sowing) and were observed until they reached the maximal adult size. A reduction of the plant size was observed for PCZ added at the highest concentration of 30 μM (T.test *p* <0.01). The other PCZ concentrations or other compounds tested had either no effect or no clear effect (T. test *p* <0.05). In particular, no increase of plant size was observed under these conditions regardless of the epiBL or Bikinin concentration tested except possibly a small increase with 5 μM Bikinin (T.test *p* <0.05).

EpiBL, Bikinin and PCZ impacted the growth response of wheat at various degrees depending on the concentration and the method of application of the compound as well as the developmental stage of the plant suggesting that BR signaling is involved in wheat growth.

### Role of BR in early embryonic development

GSK3/SGG kinases are involved in embryonic patterning and axis formation in vertebrates and invertebrates [36–38]. In comparison, little is found in the literature about the role of these kinases in plant embryonic patterning. The current study indicates that *TaSKs* were expressed in wheat embryos and were involved in BR signaling. As a first step towards understanding their function in this crucial developmental phase, we evaluated whether and to which extent BR signaling regulates *Poaceae* resp. *Triticum aestivum* embryonic development.

An *in vitro* culture system has been established in a previous study that enables a normal *in vitro* development of embryos excised from their kernels as early as at the globular stage [39]. Up to 150 μm, the embryo is globular with a hemispherical embryo-proper and a suspensor (Additional file 5, A). At 150–160 μm, the shift to bilateral symmetry is initiated with the differentiation of a scutellum and a shoot apical meristem. This shift from radial to bilateral symmetry is a major step in the embryonic development leading to the differentiation of the two major embryonic structures that are (1) the scutellum i.e., the single cotyledon of the monocotyledons and (2) the embryonic axis containing the shoot and root meristems. Over a size of 160 μm, the embryo shows a clear bilateral symmetry with a shoot meristem and a scutellum growing both axially and laterally to a shield-like structure attached to one side of the embryonic axis (Additional file 5, B-F). Gradually the shoot meristem is covered by leaf primordia and enclosed in a coleoptilar ring (Additional file 5, D -F). The root meristem differentiates embedded in the lower part of the embryo.

Embryos excised from their kernels at different developmental stages were grown *in vitro* on media supplemented with either epiBL, Bikinin or PCZ. A distinction has been made between embryos that underwent or were in the process to undergo the shift to bilateral symmetry (≥160μm) from those that had a radial shape (up to 150 μm) at the onset of treatment. An insight is thereby gained into the effect of these compounds during three major phases of embryonic patterning i.e., before and during differentiation of the shoot meristem and the scutellum, as well as during their subsequent development.

**Fig. 9:**
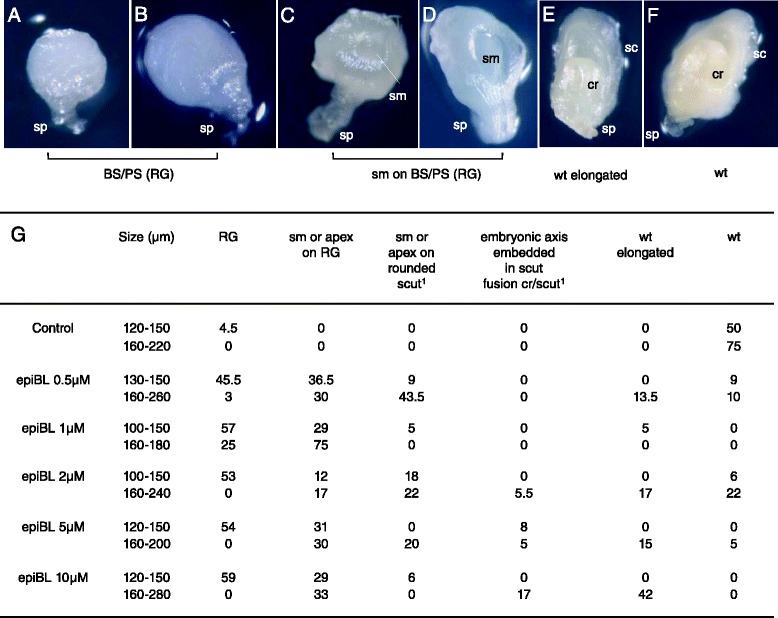
EpiBL treatment of wheat embryos. Embryos were isolated out of the kernel at sizes ranging from 100 to 280 μm in diameter, most of them having a diameter of 120 to 180 μm. They were then grown *in vitro* on media supplemented with 0 to 10 μM epiBL. The phenotypes of the *in vitro* grown embryos were observed after 7 to 8 days of culture and grouped into classes. Photographs of representative phenotypes after 7/8 days of culture were added directly to the figure (**a**-**f**, ^1^photographs not shown). Reported numbers in (**g**) are percentages of defined phenotypes per growing embryos. In at least 4 independent experiments, a total of 62 growing embryos were observed in control conditions, 41 for 0.5 μM epiBL, 33 growing embryos for 1 μM epiBL, 35 in the case of 2 μM epiBL, 34 for 5 μM epiBL and 29 in the case of 10 μM epiBL. apex: differentiation of a leaf primordium or/and a coleoptilar ring (both in most cases abnormal) in addition to a shoot meristem; cr: coleotilar ring; RG: Radial Growth (BS: Ball-Shaped or PS: Pear-Shaped); sc or scut: scutellum; sm: shoot meristem; sp: suspensor; wt: embryo having a wild type phenotype. The percentage of indifferentiated, abnormal or early arrested embryos for a given concentration and a given embryo size range is obtained by subtracting the sum of all indicated percentages to 100 %

Most embryos isolated at sizes of up to 150 μm and then subjected to epiBL treatment developed into radial growth (RG) structures (Fig. 9a, b, g). RG structures were either ball-shaped (BS) or pear-shaped (PS) (Fig. 9a and b). These embryos grew “radially” but did not undergo a shift to bilateral symmetry. In addition, a lower (except for 0.5 μM EpiBL) but nevertheless significant percentage of embryos differentiated a shoot meristem on a RG structure, in some cases even on a rounded scutellum (Fig. 9c, d and g). In these cases, a shift to bilateral symmetry occurred even though neither the differentiation of the shoot apex nor the one of the scutellum were normal. When the embryo-proper size at the moment of isolation reached 160 μm or more, i.e., the shift to bilateral symmetry was initiated before treatment, a shoot meristem developing on a RG structure or on a rounded scutellum was observed in the majority of cases (Fig. 9c, d and g). When isolated at embryo-proper sizes of 180 μm or more, a substantial number of embryos were elongated but apparently normal (Fig. 9e and g).

**Fig. 10:**
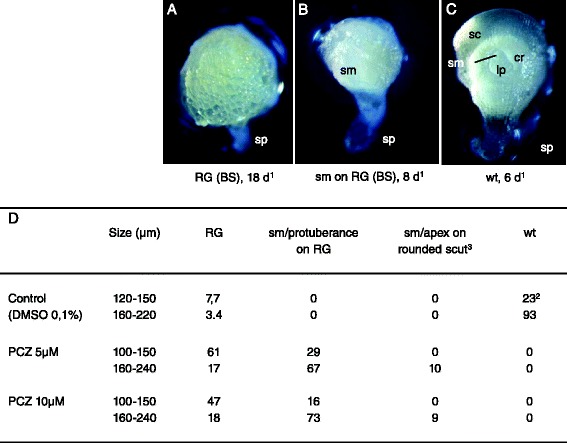
Propiconazole treatment of wheat embryos. Embryos were isolated out of the kernel at an embryo-proper diameter of 100 to 240 μm, most of them having a diameter of 120 to180 μm. The phenotypes of the *in vitro* grown embryos were observed at 5 to 28 days of culture and grouped into classes. Photographs of representative phenotypes were added directly to the table (**a**-**c**, ^3^photograph not shown). Reported numbers in (**d**) are percentages of defined phenotypes per growing embryos. In 5 independent experiments, 42 growing embryos were observed in control conditions (0.1 % DMSO), 61 in the case of 5 μM PCZ, 52 for 10 μM PCZ. ^1^days of culture. ^2^fewer control embryos isolated at sizes of 120–150 μm were developing *in vitro* into normal wild type embryos due to a higher amount of abnormal, undifferentiated or early arrested embryos. Cr: coleoptilar ring; RG: Radial Growth (BS: Ball-Shaped or PS: Pear-Shaped); sc or scut: scutellum; sm: shoot meristem; sp: suspensor; wt: embryo having a wild type phenotype. The percentage of indifferentiated, abnormal or early arrested embryos for a given concentration and a given embryo size range is obtained as in Fig. 9

The two most severe phenotypes observed upon treatment with epiBL were also observed for the majority of embryos treated with PCZ (Fig. 10). Indeed, when isolated out of their kernels at an embryo-proper size ranging from 100 to 150 μm, most embryos developed into RG (BS or PS) structures (Fig. 10a and d). When isolated with a size of 160 μm or over, the most frequent phenotype observed was a RG structure bearing a shoot meristem or a shoot meristem-like-protuberance (Fig. 10b and d). Prolongated culture on PCZ supplemented media led in many cases to the disappearance of a morphologically visible shoot meristem (data not shown).

**Fig. 11:**
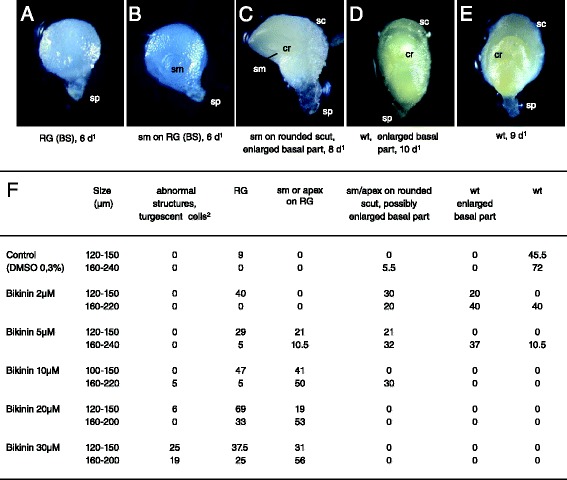
Bikinin treatment of wheat embryos. Embryos were isolated out of the kernel at sizes ranging from 100 to 240 μm in diameter, most of them having a diameter of 120 to 180 μm. The phenotypes of the *in vitro* grown embryos were observed between 4 and 13 days of culture. Photographs of representative phenotypes were added directly to the figure (**a**-**e**, ^2^photograph shown in additional file 6). Reported numbers in (**f**) are percentages of defined phenotypes per growing embryos. In three independent experiments, a total of 29, 20, 33 37, 31 and 32 growing embryos were observed respectively for the control, 2, 5, 10, 20 and 30 μM Bikinin. ^1^ days of culture. apex: differentiation of a leaf primordium or/and a coleoptilar ring (both in most cases abnormal) in addition to a shoot meristem; cr: coleotilar ring; RG: Radial Growth (BS: Ball-Shaped or PS: Pear-Shaped); sc: scutellum; sm: shoot meristem; sp: suspensor; wt: embryo having a wild type phenotype. The percentage of indifferentiated, abnormal or early arrested embryos for a given concentration and a given embryo size range is obtained as in Fig. 9

A range of phenotypes with increasing severity was observed upon Bikinin treatment tested at concentrations of 2 to 30 μM (Fig. 11). At 2 and 5 μM Bikinin, most embryos isolated at older developmental stages (160 to 240 μm) showed either a wild-type phenotype or comparatively milder phenotypes (Fig. 11c, d, e and f). The latter embryos were either wild-type but had an enlarged basal embryonic region, or exhibited a shoot meristem/apex on a rounded scutellum with a potentially enlarged basal embryonic region (Fig. 11c, d). At the same concentrations, a high number of embryos isolated at younger stages (120 to 150 μm) developed, in addition to milder phenotypes, severe phenotypes such as RG or shoot meristem/apex on RG (Fig. 11a-d, f). At higher Bikinin concentrations (10–30 μM), the vast majority of embryos displayed the latter two phenotypes (Fig. 11a, b, f). The younger embryos at the moment of isolation developed rather into RG while the older rather a shoot meristem/apex on RG. Many embryos had highly turgescent cells at higher Bikinin concentrations often leading after some time to destructuration. Thus, at 30 μM Bikinin, 19-25 % embryos were even developing into completely abnormal structures with highly turgescent cells indicating that higher concentrations of the compound were damaging for the embryos (Fig. 11f; Additional file 6).

Sporadically embryos subjected to an epiBL or a Bikinin treatment showed a duplicated embryonic axis and an additional ectopic scutellum (data not shown).

In conclusion, interfering with BR signaling or changing BR levels was impairing embryonic axis and scutellum differentiation.

## Discussion

A phylogenetic analysis of land plant GSKs pointed out that wheat TaSK1-A,B,C and TaSK2-A,B,C belong to clade II of plant GSKs [8]. The *Arabidopsis* members of clade II, ASKiota, ASKdzeta and BIN2, are all three involved in BR signaling [9–12]. Several lines of evidence strongly suggest that TaSKs were orthologous of BIN2 and were involved in BR signaling. First, expression in *Arabidopsis* of *TaSK1-A.2-1* and *TaSK2-A.2-1* having a *bin2-1* like gain-of-function mutation led to phenotypes closely resembling those of *Arabidopsis bin2-1* lines and *Arabidopsis* BR-signaling or BR-deficient mutants [9–11]. Second, BR target gene expression was deregulated in *TaSK1-A.2-1* and *TaSK2-A.2-1* dwarf lines as expected for phenotypes due to an impaired BR signaling. Third, the severe dwarf *TaSK.2-1* phenotype was rescued partially by Bikinin, an inhibitor of TaSK kinase activity. This rescue was accompanied with changes of *SAUR, BAS1* and *CPD* transcript levels. In summary, expression of TaSK1 and TaSK2 in *Arabidopsis* led to responses similar to those of *Arabidopsis* BR signaling intermediate BIN2. These data strongly suggest that both wheat homologs were negative regulators of BR signaling. Consequently, a function of GSK clade II in BR signaling is proposed to be evolutionary conserved between *Poaceae* and *Brassicaceae* confirming our phylogenetic data.

Bikinin was identified in a chemical genetics approach as a strong activator of BR signaling [27]. This compound is a strong chemical inhibitor of group I and II *Arabidopsis* GSKs [27]. Total inhibition (1–2 % residual activity) is reported for clade II kinases including BIN2 while some residual activity (6–8 % residual activity) is observed for clade I kinases [27]. In addition, Bikinin inhibits 80 % of the activity of group III ASKtheta bringing the number of inhibited ASKs up to 7 out of 10 [27]. Bikinin was inhibiting all three wheat GSKs tested: clade II TaSK1 and TaSK2 as well as clade I TaGSK1. Inhibition was very strong for TaSK2 (5 % residual activity at 15μM Bikinin) to strong for TaSK1 and TaGSK1 (significantly less than 30 % residual activity for TaSK1 at 30 μM Bikinin). The specific residues M115, Y117 and Val118 are important for the binding of Bikinin to the ATP binding pocket of BIN2 [27]. This motif is also present in the two other clade II ASKs [27]. TaSK1 and TaSK2 were inhibited at different levels although they possessed both this MEYV motif [8]. In addition, inhibition levels of TaSK1 and TaGSK1 were similar even-though TaGSK1 has a different motif as TaSK1 namely the motif LEYV present in all clade I ASKs and in clade III ASKtheta. Identification of other wheat GSKs will be beneficial to study further the relevance of this motif for Bikinin inhibitory activity on *Poaceae* GSKs.

Treatment of wheat seedling with epiBL, PCZ, and Bikinin indicated that seedling growth was regulated by BR signaling. Growth response was depending on the method of application, on the concentration of the compound applied and on the plant developmental stage. When added to the culture medium of germinating seeds, epiBL, PCZ and Bikinin inhibited the growth of the aerial part of the seedling in a dose dependent manner. Although this response contrasts with the growth response of *Brassicaceae* resp*. Arabidopsis* [27, 29], other studies reported a similar response in *Poaceae* [32, 40]. EpiBL is decreasing the growth of wheat seedlings when applied through the roots [32]. This reduced growth is suggested to be due to an overaccumulation in the plant of exogenously applied BR [32]. Indeed application of 2 μM 24-epibrassinolide via the roots or seeds increases 3 to 4 times the level of 24-epiBL in the plant [32]. Similarly, reports indicated that BR oversensitivity does not always result in stem elongation and that a reduction of rice stem growth can be induced by either insensitivity or oversensitivity to BR [40]. Therefore, the height reduction of wheat seedling observed when the compounds were uptaken *via* the roots may be due to an oversensitivity to BR. Foliar application of epiBL is reported to stimulate the growth of the upper part of the wheat seedling [32]. By contrast, in our experimental conditions, foliar spraying of epiBL or Bikinin did not result in a noteworthy increase of wheat seedling height while as expected the aerial seedling growth was significantly reduced in the presence of PCZ. However, epiBL was increasing the bending angle between first leaf blade and sheath while PCZ treatment was decreasing this angle. Such a response is also observed in rice. Indeed, adding exogenously BRs and increasing BR biosynthesis or signaling enhances rice lamina joint bending whereas BR insensitive or deficient plants have erect leaves [34, 35, 41]. Bikinin was affecting wheat laminar joint inclination at concentrations over 30 μM. These concentrations although being very efficient were higher than the ones leading to a size reduction of wheat seedlings (*via* root uptake) and the ones impacting embryonic development. Foliar spraying cannot be directly compared to uptake over several days *via* the seedling roots or from the medium by tiny embryos. Differences in tissue permeability as well as inactivation of the compound *in planta* [42] may require higher concentrations if the uptake occurs *via* the leaves over a limited time. PCZ was used as alternative to BRZ that had little effect on wheat seedling growth. PCZ is proposed to be a specific and potent inhibitor of BR biosynthesis [31, 43]. PCZ inhibits fungal sterol synthesis by binding to lanosterol 14R-demethylase (CYP51A1) [31]. Reports indicate that the triazole may inhibit activity of obtusifoliol 14-alpha-demethylase in plants [43]. PCZ may therefore alter BR metabolic pathway by affecting sterol biosynthesis upstream of BR synthesis. However, *Arabidopsis* seedlings treated with PCZ phenocopied BR biosynthetic mutants and dwarfism was induced in maize seedling [31]. In addition, although triazole derivatives may affect in some cases GA biosynthesis, the expression levels of gibberellic acid regulated genes was reported as not affected by PCZ application while the expression of BR regulated genes was specifically impacted [31]. In both cases biosynthesis genes were analyzed.

*TaSK1* and *2* have been isolated in the screen of an embryonic cDNA library [8]. Little is known about the role of GSKs in plant embryonic patterning. In vertebrates GSK3/SGG orthologs are negative regulators of the embryonic dorsoventral axis formation [44]. A deficiency for the activity of this protein kinase leads to the ectopic formation of a secondary body axis in *Xenopus laevis* embryo [37, 44]. In Invertebrates such as *Hydra*, inhibition of the HyGSK-3 activity results in body column adopting features of the head organizer [38]. As a consequence, ectopic heads and tentacles differentiate on the body column. Interestingly, wheat embryos are differentiating additional ectopic shoot meristems or embryonic axes as well as supernumerary scutella upon N-1-naphthylphthalamic acid (NPA) or quercetin treatment, both being auxin polar transport inhibitors [45]. Multiple meristems and scutella were observed only very sporadically upon EpiBL and Bikinin treatments. Instead, deregulating BR signaling in either way, inhibition or enhancement, impaired embryonic axis and scutellum differentiation leading in extreme cases to radial growth structures that were unable to undergo the shift to bilateral symmetry. Interestingly radial growth phenotypes are also observed upon auxin treatment [46]. Previous studies reported that directed cell elongation and scutellum growth are required for normal wheat embryonic patterning [47, 48]. Thereby, a directed transport of auxin is taking place towards epidermal cells of the scutellum. Accumulated auxin is proposed to increase PM H + -ATPase expression level resulting in an apoplastic acidification of the cells in these regions, a process proposed to contribute to cell wall loosening and elongation of the scutellum [47, 48]. Present observations raise the question whether the differentiation of the scutellum and the embryonic axis may depend upon directed cell elongation regulated by auxin *and* BR pathways. Interfering with the production of BR (PCZ) may hamper elongation of embryonic cells. Boosting BR signaling (epiBL, Bikinin) may lead to cell elongation in general rather than to the elongation of specific cells. The consequence in both cases may be that neither an embryonic axis nor a scutellum is able to differentiate or to differentiate properly leading to a range of radial-growth-like structures. A possible role for BRs in embryonic patterning has also to be discussed in the light of emerging evidence that BRs are apparently not themselves transported over long distances but may alter the transport of auxin [27, 49]. Interactions between BR and auxin signaling, as well as an interdependence of their transcriptional response have been reported in the literature [50]. In particular, Bikinin and BL down-regulate genes involved in the auxin pathway such as the auxin efflux regulator *PIN7* involved in processes underlying *Arabidopsis* embryonic development [27, 51]. Furthermore, BIN2 acts as a molecular link between auxin and BR signaling [52]. Information on a role of BR signaling in plant embryonic patterning is so far limited. However, recent evidences suggest a connection to auxin signaling in the control of embryonic root initiation. ACTIVATION-TAGGED BRI1-SUPPRESSOR1 (ATBS1)/TARGET OF MONOPTEROS 7 (TMO7) is an atypical basic helix loop helix (bHLH) transcription factor and is described as a positive regulator of BR signaling [53]. *ATBS1/TMO7* has been identified as a direct target of the auxin-dependent transcription factor MONOPTEROS and is involved in the MP-dependent hypophysis specification and embryonic root initiation in *Arabidopsis* [54]. Interestingly, TMO7 is moving at the globular stage from its site of synthesis in adjacent group of embryo cells into the hypophysis precursor [54]. Embryonic root specification is thus depending on a MP-dependent intercellular movement of TMO7 in addition to MP promoted PIN-FORMED 1-dependent auxin transport [54]. Furthermore, BES1 interacting Myc-like protein 1 (BIM1), another bHLH protein implicated in BR signaling, is also contributing to the control of *Arabidopsis* embryonic patterning [23, 55]. BIM1 interacts with two AP2 transcription factors, DORNRÖSCHEN (DRN) and DORBRÖSCHEN-LIKE (DRNL), both controlling *Arabidopsis* embryonic patterning [55, 56]. Loss of function of *BIM1* leads to cell division defects in the hypophyseal region and defects in the cotyledon ontogeny [55]. As DRN is involved in auxin signaling, interaction between BIM1 and DRN may be a point of hormonal crosstalk in embryonic patterning [55, 56].

BR signaling has in addition a role in root growth after germination. Indeed, BRI1 activity in the root epidermis controls the size of the root meristem [57]. Interestingly, BR –mediated control of meristem size is not associated with changes in expression levels of *PIN1, PIN7* and *PIN3* [57]. In addition, BR signaling has been shown recently to regulate stem cell quiescence in the *Arabidopsis* primary root [58].

A deeper insight into the role of BR signaling in embryonic development would include the ability to target and hamper solely BR biosynthesis. As already mentioned, PCZ may target sterol biosynthesis upstream of BR biosynthesis [31, 43]. Sterol biosynthetic mutants such as *fackel* and *hydra* 1/2 are impaired in embryonic patterning [59, 60]. However, *fackel* mutants accumulate low levels of BRs [61]. Mutant seedlings respond but cannot be rescued by BRs nor can the mutants be rescued by bulk sterols [59, 60]. Therefore, studies performed in *Arabidopsis* [62] may be complemented by the analysis of a broader range of BR-deficient mutants, and inhibitors specific for BR synthesis and efficient for *Arabidopsis* such as BRZ.

## Conclusions

Little is known about the function of GSK-like kinases in *Triticum aestivum.* TaGSK1 and TaSK5, both members of clade I, have so far been investigated and reported to be involved in abiotic stress tolerance [28, 63]. The present study provides strong evidence for an involvement of two GSK homologs, TaSK1 and 2, in brassinosteroid signaling. BIN 2.1-like gain-of-function mutation of TaSK1 or 2 expressed in *Arabidopsis thaliana* resulted in dwarf phenotypes reminiscent of BR signaling mutants. BR downstream target gene expression was impacted in *TaSKs.2-1* dwarf lines. Bikinin, shown here to inhibit TaSK kinase activity, was able to rescue partially the severe dwarfs. This rescue was associated with changes of BR downstream gene expression. Consequently, these wheat kinases expressed in *Arabidopsis* led to responses very similar to those of *Arabidopsis* autologous kinase BIN2. A function for GSK in BR signaling is therefore proposed to be evolutionary conserved between Liliopsida resp. *Poaceae* and eudicotyledons resp. *Brassicaceae.* Our previous phylogenetic analysis that classified TaSKs in clade II, the *Arabidopsis* members of which are all involved in BR signaling corroborates these findings. In plants, GSKs appear to be primarily involved in BR signaling although crosstalks to other hormonal signaling pathways have been reported, illustrating the complexity of signaling networks underlying plant development.

Limited information on the role of BR signaling in wheat development made inescapable its investigation in a critical developmental phase such as embryonic development. The present investigations indicate that interfering with BR signaling or changing BR levels by means of treatments with chemical compounds such as Epibrassinolides, Bikinin or Propiconazole impaired embryonic axis and scutellum differentiation. These findings lay the foundation to explore a potential function of TaSKs in wheat embryonic development and in particular to evaluate whether they may be a molecular link between auxin and BR pathway in these developmental processes.

## Methods

### *Arabidopsis* plant material and growth

*Arabidopsis thaliana* ecotype Columbia (Col-0) and the different transgenic lines generated in this study were grown at 21 °C under white light (16h light/8h dark cycles) either on ½ Murashige and Skoog (MS) medium containing 1 % sucrose and 25 μg/ml hygromycin or in soil.

### Generation of *Arabidopsis thaliana* lines expressing GFP-TaSK1.2-1 and GFP-TaSK2.2-1

*TaSK-1A and TaSK-2A* [8] were cloned into plasmid *pGEN016* to generate *eGFP:TaSK1-A* and *eGFP:TaSK2-A* fusions. Plasmid site directed PCR mutagenesis was used to introduce the specific amino acid exchange E263K within the TREE motif of TaSKs. This site directed mutation corresponds to *bin2-1* gain of function mutation [10]. *Pfu* DNA polymerase (Fermentas) in combination with primers T1A-bin2.1-F/T1A-bin2.1-R and T2A-bin2.1-F/T2A-bin2.1-R were used for respectively *TaSK1* and *TaSK2* mutagenesis (Additional file 7). *eGFP:TaSK1-A.2-1* and *eGFP:TaSK2-A.2-1* fusions were recloned with their C-terminus in frame with a 6XHis tag into plasmid pCAMBIA1390_2X35S. They were driven by a double-enhancer version of the CaMV35S promoter. pCAMBIA plasmids were subsequently individually introduced in *Agrobacterium tumefaciens* strain GV3101 and *Arabidopsis thaliana* was transformed by floral dip [64]. Similar procedure was used to introduce and overexpress BIN2-1 in *Arabidopsis.*

The presence of transgenes in the lines was confirmed using specific *TaSK1* and *TaSK2* primers (Additional file 7).

### *In vitro* kinase activity assays

TaSK1, TaSK2, BIN2 and TaGSK1 (GenBank: AF525086) full length proteins were cloned with their N-terminus in frame with a Gluthatione-S-Transferase (GST) tag [8].

GST-TaSK1, GST-TaSK2, GST-BIN2, and GST-TaGSK1 fusion proteins were overexpressed in *E. Coli*. and affinity purified in native conditions on Gluthatione Sepharose 4B resin.

1.5 μg of each of the purified fusion proteins was incubated with or without Bikinin (15 or 30 μM) for 4 h at room temperature in a total volume of 15 μl.

*In vitro* kinase reactions were performed by adding to each of these solutions, 15 μl of the kinase activity buffer (20 mM HEPES, pH 7.4, 20 mM MgCl2, 1 mM DTT, 50 μM non- radioactive ATP), containing 4 μCi ATP γ^32^P and 1.5 μg of bovine myelin basic protein (MBP). The reactions were incubated at room temperature for 45 min. They were stopped by adding 10 μl of SDS-PAGE loading buffer. After denaturation at 95 °C for 1 min, protein phosphorylation was analyzed by autoradiography after migration on a 12 % SDS/PAGE gel.

### Bikinin rescue experiments

*TaSK1-A.2-1* and *TaSK2-A.2-1* transgenic lines were grown at 21 °C under white light (16h light/8h dark cycles) on ½ Murashige and Skoog (MS) medium containing 1 % sucrose and 25 μg/ml hygromycin. After 15–18 days of culture, severe dwarf phenotypes were selected and transferred on ½ MS medium containing 1 % sucrose and 30 μM Bikinin (4-[(5-bromo-2-pyridinyl)amino]-4-oxobutanoic acid, ChemBridge Corporation, USA) or 0.3 % DMSO for 7–8 days under the same light and temperature conditions. Presence for the transgenes in the severe dwarf lines was confirmed using specific primers for either *TaSK1* or *TaSK2* (Additional file 7). Columbia seedlings were grown under the same light and temperature conditions on ½ MS medium containing 1 % sucrose for 14 days and then transferred on the same medium supplemented with either 30 μM Bikinin or 0.3 % DMSO for 7 days.

### Quantitative Real-Time Polymerase Chain reaction

RNA was extracted according to Oñate-Sánchez and Vicente-Carbajosa (2008) [65]. cDNA was prepared from total RNA with TaqMan® Reverse Transcription Reagents (Applied Biosystems) and analyzed on a Step One Plus LigthCycler (Applied Biosystems) with SYBR Green reagents (MESA Green qPCR MasterMix Eurogenetec) according to manufacturer’s instructions.

Transcripts of *TaSK1, TaSK2* and BR signaling target genes were quantified using specific primer pairs (Additional file 7). Selected target genes were *SAUR-AC1* [TAIR: AT4G38850.1], *CPD* [TAIR: AT5G05690] and *BAS1* [TAIR: AT2G26710]. All individual reactions were done in triplicate (technical replicates). Data were analysed with StepOne™ Software v2.2.2 (Applied Biosystems). Expression levels were normalized to those of *UBQ10* [TAIR: AT4G05320] or *EF-1alpha* [AT5G60390]. Relative quantification of gene expression was performed using the standard curve method.

### Semi-quantitative RT-PCR analysis

Total RNA was extracted from different wheat tissues including stem, root, leaves (young and adult), embryo, endosperm, seed and flowers using TRIzol® Reagent (Invitrogen). One μg of total RNA (DNA free) was used for reverse transcription performed by means of the SuperScript™ II Reverse Transcriptase (Invitrogen).

The gene specific primers SF27/SR28 were used to amplify *TaSK1* while the primers SF34/SR34 were used to amplify *TaSK2* (Additional file 7). Amplification of tubulin gene was used as RT-PCR reference (Additional file 7). A digestion of PCR products using *BglII* endonuclease confirmed the specificity of *TaSK1* and *TaSK2* amplification (data not shown).

### Hormone and inhibitor treatments of wheat seedlings and plants

Wheat cv Sonora seeds were germinated and cultured for 6 days on ½ Murashige and Skoog medium containing 1 % sucrose at 25 °C under white light (16h light/8h dark cycles). This medium was supplemented with either epibrassinolide (epiBL - Sigma-Aldrich E1641; concentration range: 0.5 to 10 μM), Bikinin (Sigma-Aldrich SML 0094; concentration range: 5 to 30 μM), Brassinazole (BRZ – TCI B2829; concentration range: 2 to 30 μM) or Propiconazole (PCZ – Syngenta Tilt 250 EC; concentration range: 1 to 30 μM). Only seeds having germinated after 2 days were taken in account for length measurement. The length of the upper part of the seedling (without roots) was measured after 6 days.

Seeds were also germinated on filter paper that has been previously soaked with water and transferred after 2 days on vermiculite. They were grown in the incubator under the same conditions as the *in vitro* grown seedlings. Under these conditions, germination of the seeds and initial growing phase of the seedlings was synchronous. The aerial part of the seedlings was then sprayed 4 times (at 6, 7, 8, 9 days after sowing) with either water, 0.25–0.4 % DMSO, 2–10 μM epiBL, 5–30 μM PCZ or 10-80 μM Bikinin. Tween 20 was added at 0.02 % as surfactant to the different solutions. The length of the seedling without the root system was measured 14 days after sowing. The first leaf inclination was measured at the same time point.

In addition, seeds were germinated and grown in soil in the greenhouse under 16h light/8h dark cycles and 18/20 °C day - 12/15 °C night temperatures. The aerial part of the plants were sprayed 3 times with either water, 0.05 to 0.3 % DMSO, 2 to12 μM epiBL, 5 to 30 μM PCZ and 5 to 30 μM Bikinin. Tween 20 at 0.02 % was added as surfactant to the different solutions. Length of each plant without the root system was measured at the time of the first spraying (15–22 days after sowing) and once the maximum plant size (50–55 days after sowing) was reached.

### Hormone and inhibitor treatment of wheat embryos

Wheat Cv Sonora were grown in the greenhouse under 16h light/8h dark cycles and 18/20 °C day - 12/15 °C night temperatures. Embryos were isolated and grown *in vitro* as described by Fischer and Neuhaus (1995) and Fischer et al., (1997) [39, 45]. EpiBL was added to the culture media at concentrations ranging from 0.5 to 10 μM, PCZ at concentrations ranging from 5 to 10 μM and Bikinin at concentrations ranging from to 2 to 30 μM.

## Availability of supporting data

All supporting data are included as additional files

## Additional files

Additional file 1:
**BR target gene expression levels in** ***TaSK1.2-1*** **transgenic lines.** (PDF 112 kb) Additional file 2:
**Effect of Bikinin on BR target gene expression levels in** ***TaSK1.2-1*** **severe dwarf lines.** (PDF 235 kb) Additional file 3:
**Response of** ***in vitro*** **grown wheat seedlings to low epiBL concentrations.** (PDF 87 kb) Additional file 4:
**Response of wheat plants grown under greenhouse conditions to epiBL, Bikinin, and PCZ foliar spray.** (PDF 96 kb) Additional file 5:
**Normal** ***in vitro*** **development of wheat embryos.** (PDF 64 kb) Additional file 6:
**Bikinin-treated wheat embryo.** (PDF 16 kb) Additional file 7:
**List of primers.** (PDF 74 kb)

## Abbreviations

ASK: *Arabidopsis* SHAGGY-related protein kinase
ATBS1: ACTIVATION-TAGGED BRI1-SUPPRESSOR1
*BAS1*: *phyB activation-tagged suppressor 1*
BES1: BRI1-EMS-SUPPRESSOR1
bHLH: basic helix loop helix
BIM1: BES1 interacting Myc-like protein 1
BIN2: BRASSINOSTEROID INSENSITIVE 2
BL: brassinolide
BR: brassinosteroid
BRI1: BRASSINOSTEROID INSENSITIVE 1
BRZ: brassinazole
BS: ball-shaped
BSK1: BR-SIGNALING KINASE 1
BSU1: BRI1 SUPPRESSOR 1
BZR1: BRASSINAZOLE-RESISTANT1
*CPD*: *constitutive photomorphogenesis and dwarfism*
cr: coleoptilar ring
CYP51A1: lanosterol 14R-demethylase
DRN: DORNRÖSCHEN
*EF-1alpha*: *elongation factor-1alpha*
ep: embryo-proper
epiBL: Epibrassinolide
GA: gibberellin
GFP: Green Fluorescent Protein
GSK: Glycogen Synthase Kinase 3/SHAGGY-like kinase
GST: Gluthathione-S-Transferase
MP: MONOPTEROS
MPB: bovine myelin basic protein
MS: Murashige and Skoog
NPA: N-1-naphthylphthalamic acid
PCZ: Propiconazole
PIN: PIN-FORMED
PS: pear-shaped
RG: radial growth
*SAUR-AC1*: *Arabidopsis Columbia Small Auxin Up RNA gene 1*
sc: scutellum
SD: severe dwarf
SGG: SHAGGY
sm: shoot meristem
sp: suspensor
TaGSK1: *Triticum aestivum* glycogen synthase kinase - SHAGGY kinase
TaSK: *Triticum aestivum* SHAGGY-like Kinase
TMO7: TARGET OF MONOPTEROS 7
*UBQ10*: *polyubiquitin 10*
