# Supplementary material for: Two homolog wheat Glycogen Synthase Kinase 3/SHAGGY - like kinases are involved in brassinosteroid signaling
Source: BMC Plant Biol. 2015 Oct 13;15:247. doi: 10.1186/s12870-015-0617-z (PMC4604091; doi:10.1186/s12870-015-0617-z)

Additional file 1: BR target gene expression levels in *TaSK1.2-1* transgenic lines

mRNA levels of BR target genes were quantified by qRT-PCR in *TaSK1-A.2-1* lines under the same experimental conditions as those described in figure 2. Expression levels were however in this case normalized to those of *EF-1alpha*.

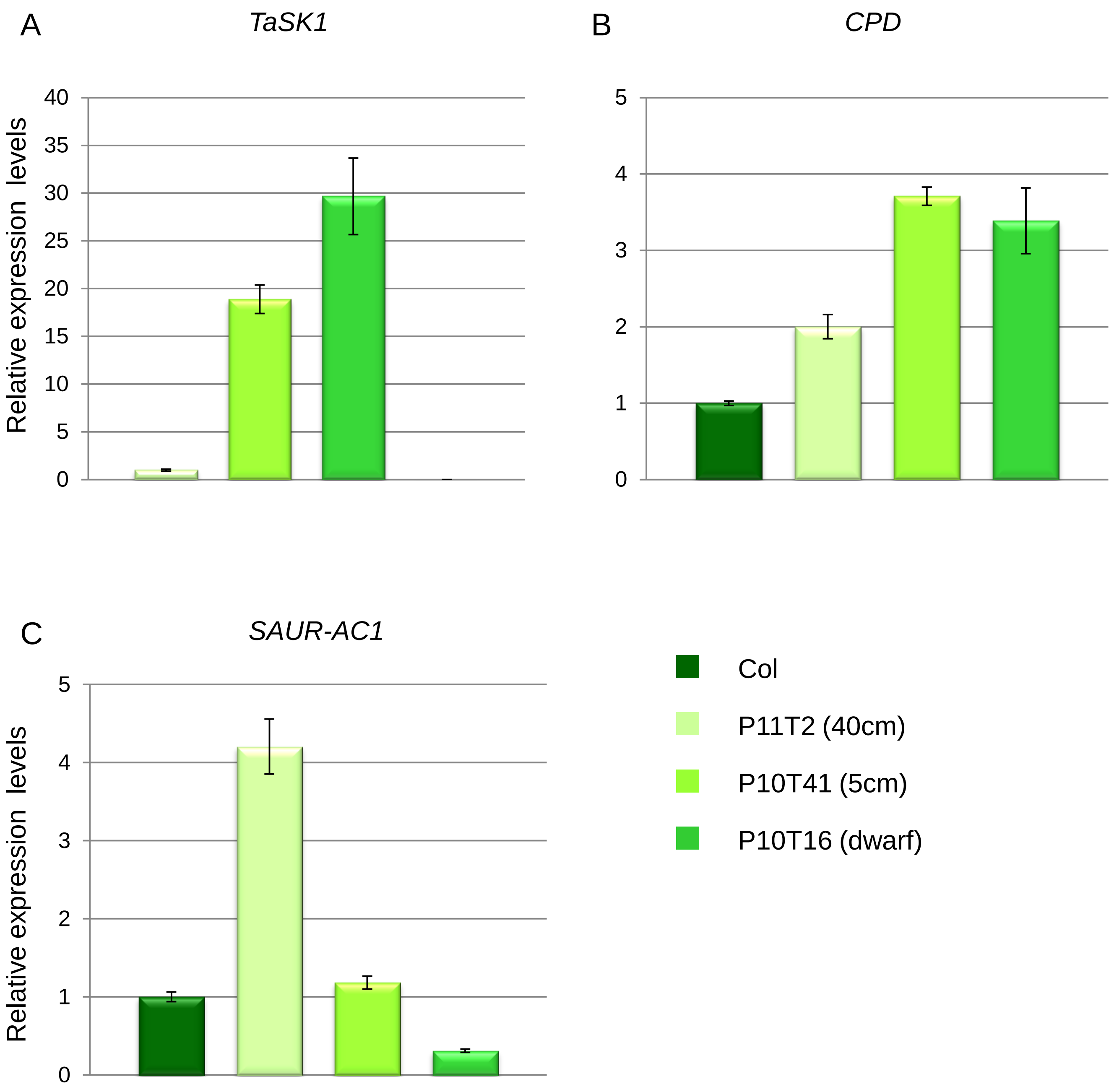

Supplement: Additional file 1: — BR target gene expression levels in TaSK1.2-1 transgenic lines. (PDF 112 kb) [file 12870_2015_617_MOESM1_ESM.pdf]
