# Supplementary material for: Two homolog wheat Glycogen Synthase Kinase 3/SHAGGY - like kinases are involved in brassinosteroid signaling
Source: BMC Plant Biol. 2015 Oct 13;15:247. doi: 10.1186/s12870-015-0617-z (PMC4604091; doi:10.1186/s12870-015-0617-z)
Supplement: Additional file 2: — Effect of Bikinin on BR target gene expression levels in TaSK1.2-1 severe dwarf lines. (PDF 235 kb) [file 12870_2015_617_MOESM2_ESM.pdf]

Additional file 2: Effect of Bikinin on BR target gene expression levels in *TaSK1.2-1* severe dwarf lines

mRNA levels of BR target genes were quantified by qRT-PCR in *TaSK1-A.2-1* severe dwarf (SD) lines under the same experimental conditions as those described in figure 5. Expression levels were however in this case normalized to those of *EF-1alpha*.

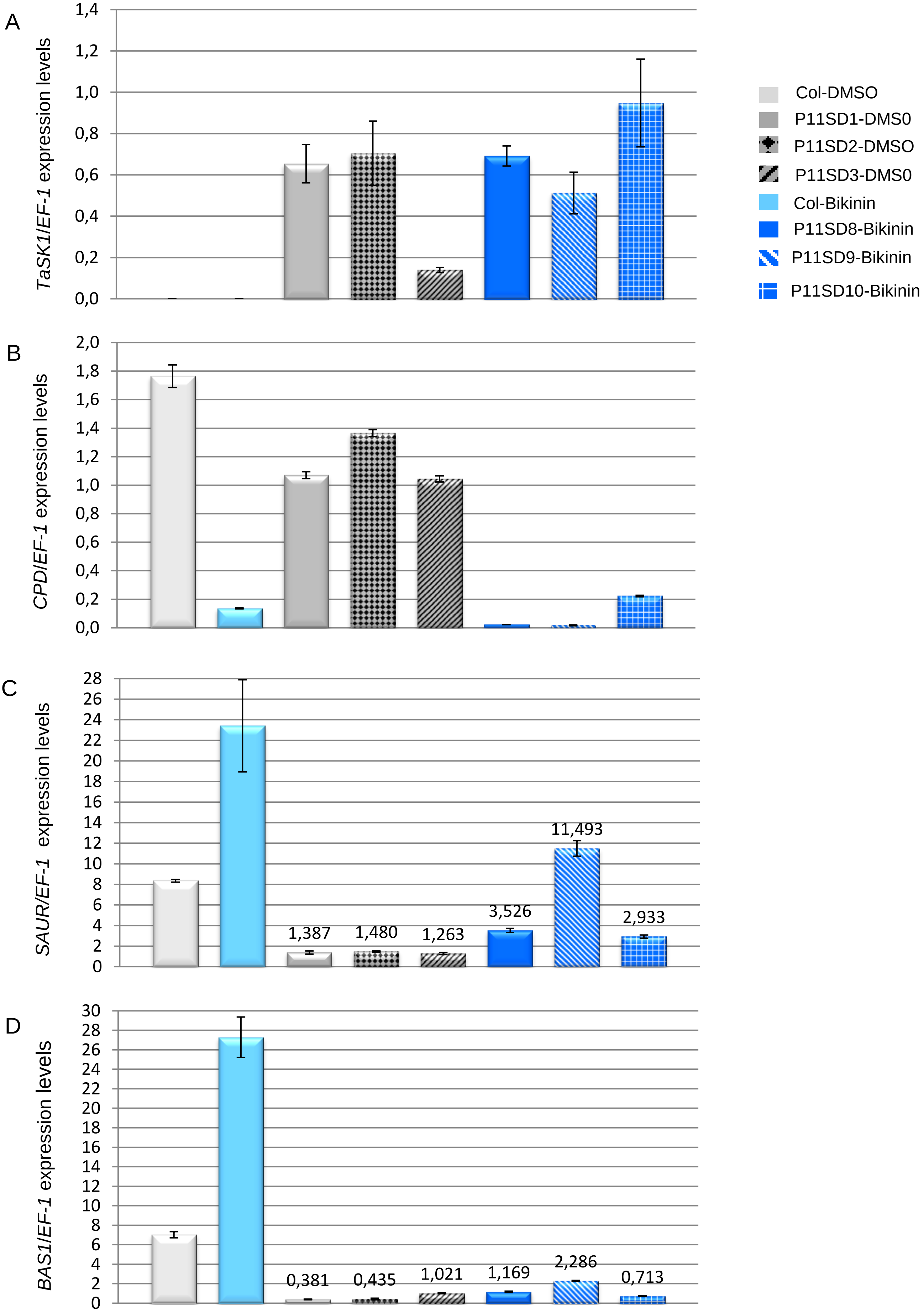
A
TaSK1/EF-1 expression levels
1,4
1,2
1,0
0,8
0,6
0,4
0,2
0,0
Col-DMSO
P11SD1-DMS0
P11SD2-DMSO
P11SD3-DMS0
Col-Bikinin
P11SD8-Bikinin
P11SD9-Bikinin
P11SD10-Bikinin
B
CPD/EF-1 expression levels
2,0
1,8
1,6
1,4
1,2
1,0
0,8
0,6
0,4
0,2
0,0
C
SAUR/EF-1 expression levels
28
26
24
22
20
18
16
14
12
10
8
6
4
2
0
1,387
1,480
1,263
3,526
11,493
2,933
D
BAS1/EF-1 expression levels
30
28
26
24
22
20
18
16
14
12
10
8
6
4
2
0
0,381
0,435
1,021
1,169
2,286
0,713
