# Supplementary material for: Two homolog wheat Glycogen Synthase Kinase 3/SHAGGY - like kinases are involved in brassinosteroid signaling
Source: BMC Plant Biol. 2015 Oct 13;15:247. doi: 10.1186/s12870-015-0617-z (PMC4604091; doi:10.1186/s12870-015-0617-z)

Additional file 3: Response of *in vitro* grown wheat seedlings to low epiBL concentrations.

Wheat seeds were sown and cultured for 6 days under white light (16hr light/8hr dark cycles) on ½ MS medium containing 1 % sucrose supplemented with 0.5 and 1 µM epiBL.
Values represent mean values and standard deviations of seedling aerial part length (n=18) measured after 6 days of culture.
T.test $p<0.01$ for 1 µM epiBL

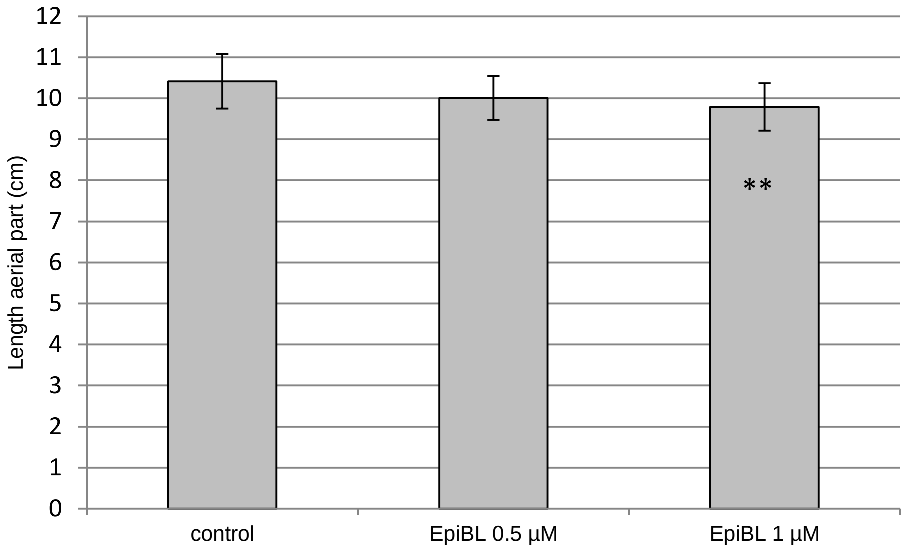

Supplement: Additional file 3: — Response of in vitro grown wheat seedlings to low epiBL concentrations. (PDF 87 kb) [file 12870_2015_617_MOESM3_ESM.pdf]
