# Supplementary material for: Two homolog wheat Glycogen Synthase Kinase 3/SHAGGY - like kinases are involved in brassinosteroid signaling
Source: BMC Plant Biol. 2015 Oct 13;15:247. doi: 10.1186/s12870-015-0617-z (PMC4604091; doi:10.1186/s12870-015-0617-z)
Supplement: Additional file 4: — Response of wheat plants grown under greenhouse conditions to epiBL, Bikinin, and PCZ foliar spray. (PDF 96 kb) [file 12870_2015_617_MOESM4_ESM.pdf]

Additional file 4: Response of wheat plants grown under greenhouse conditions to epiBL, Bikinin, and PCZ foliar spray.

Selected concentrations of epiBL, Bikinin and PCZ were sprayed 3 times on the leaves of wheat plants grown in the greenhouse. Values represent mean values and standard deviations of the difference between maximum plant size reached (50-55 days after sowing) and plant size at the time of the first spraying (15-22 days after sowing) considering only the aerial part without the root system. For each treatment or control, 10 plants were sown out except for epiBL 4 µM, Bikinin 10 µM and PCZ 10 µM for which 15 plants were sown out.
* T.test $p<0,05$ ($p=0,01144745$ for 5 µM Bikinin; $p= 0,01859155$ for 5 µM PCZ),
**T.test $p<0,01$ ($p=0,00141834$ for 30 µM PCZ)

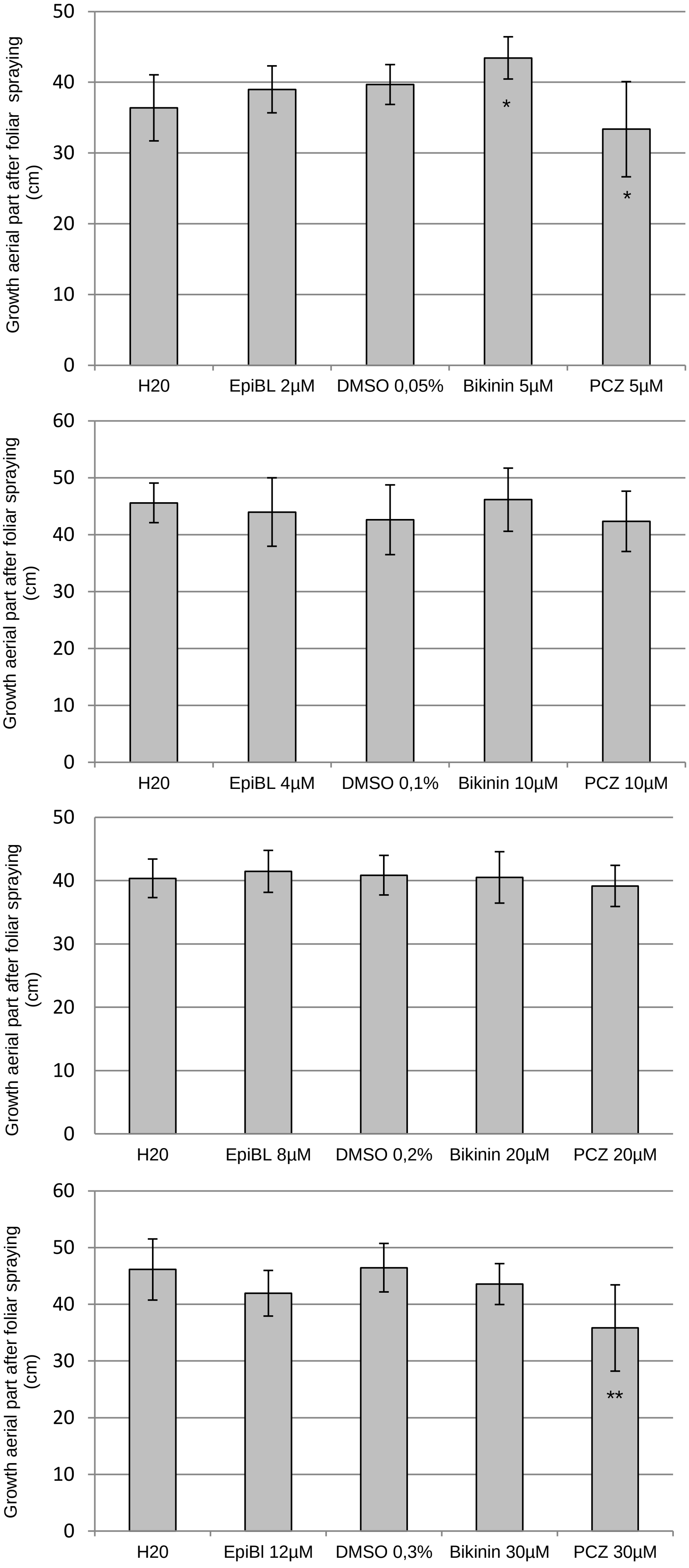
Growth aerial part after foliar spraying (cm)
0
10
20
30
40
50
H20
EpiBL 2µM
DMSO 0,05%
Bikinin 5µM
PCZ 5µM
*
*
Growth aerial part after foliar spraying (cm)
0
10
20
30
40
50
60
H20
EpiBL 4µM
DMSO 0,1%
Bikinin 10µM
PCZ 10µM
Growth aerial part after foliar spraying (cm)
0
10
20
30
40
50
H20
EpiBL 8µM
DMSO 0,2%
Bikinin 20µM
PCZ 20µM
Growth aerial part after foliar spraying (cm)
0
10
20
30
40
50
60
H20
EpiBl 12µM
DMSO 0,3%
Bikinin 30µM
PCZ 30µM
**
