# Supplementary material for: Two homolog wheat Glycogen Synthase Kinase 3/SHAGGY - like kinases are involved in brassinosteroid signaling
Source: BMC Plant Biol. 2015 Oct 13;15:247. doi: 10.1186/s12870-015-0617-z (PMC4604091; doi:10.1186/s12870-015-0617-z)
Supplement: Additional file 5: — Normal in vitro development of wheat embryos. (PDF 64 kb) [file 12870_2015_617_MOESM5_ESM.pdf]

Additional file 5: normal *in vitro* development of wheat embryos

(A) Globular embryo with an embryo-proper (ep) diameter of 140 µm at the time of isolation. (B) Embryo having a diameter of 180 µm at the time of isolation. The scutellum (sc) is growing in the axial and the lateral directions, which results in a prominent structure on one side of the embryo-proper. The swelling on the other side of the embryo-proper indicates the onset of the shoot apical meristem differentiation (sm). (C) to (F) Embryos grown *in vitro* on media free of inhibitors. (C) Embryo that differentiated a shoot meristem and a scutellum (width = 240 µm). (D), (E) The first leaf primordium (lp) is progressively overgrowing and covering the shoot meristem (sm). The shoot meristem and leaf primordia are enclosed by the coleoptile (cr). The width of embryos depicted in (D) and (E) are 340 µm and 530 µm *resp*. (F) mature embryo showing a shield-like scutellum and an embryonic axis containing a shoot (enclosed by the coleoptilar structure) and a root meristem (enclosed by the coleorhiza, not shown)(embryo width = 1100 µm).
cr: coleotilar ring; c: coleoptile; lp: first leaf primordium; ep: embryo-proper; sc: scutellum; sm: shoot meristem; sp: suspensor.

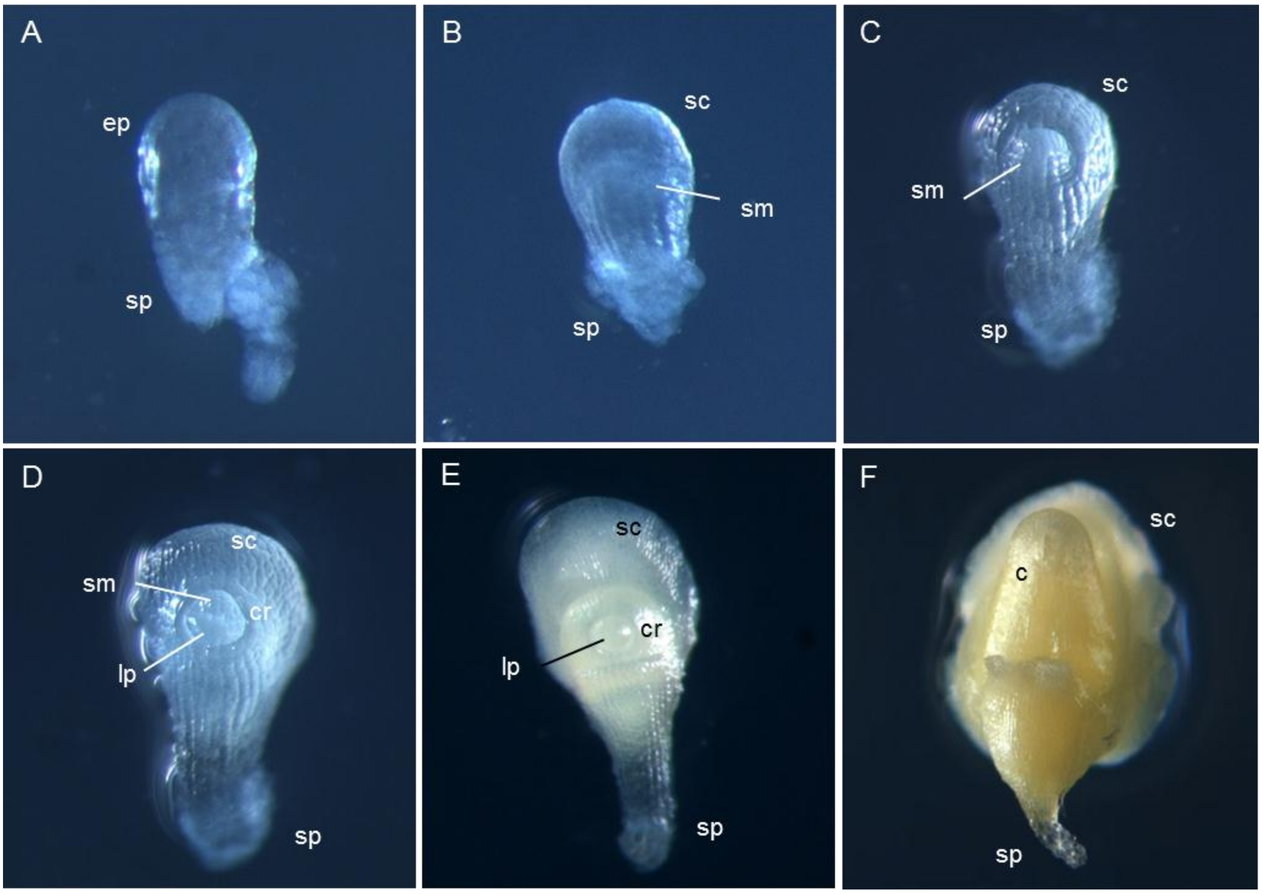
A
ep
sp
B
sc
sm
sp
C
sc
sm
sp
D
sc
sm
cr
lp
sp
E
sc
cr
lp
sp
F
sc
c
sp
