# Supplementary material for: Two homolog wheat Glycogen Synthase Kinase 3/SHAGGY - like kinases are involved in brassinosteroid signaling
Source: BMC Plant Biol. 2015 Oct 13;15:247. doi: 10.1186/s12870-015-0617-z (PMC4604091; doi:10.1186/s12870-015-0617-z)

Additional file 6: Bikinin-treated wheat embryo

Photograph depicts a globular wheat embryo (isolated at 140 µm) grown for 7 days on media supplemented with 30 µM Bikinin. This embryo developed highly turgescent cells.

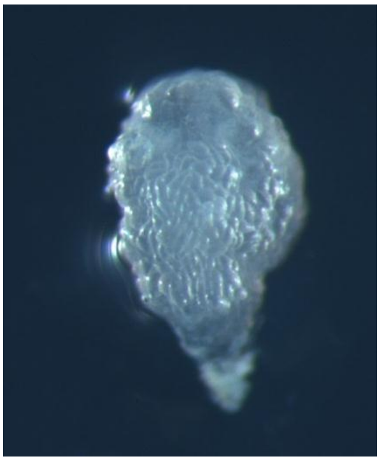

Supplement: Additional file 6: — Bikinin-treated wheat embryo. (PDF 16 kb) [file 12870_2015_617_MOESM6_ESM.pdf]
