# Supplementary material for: Two homolog wheat Glycogen Synthase Kinase 3/SHAGGY - like kinases are involved in brassinosteroid signaling
Source: BMC Plant Biol. 2015 Oct 13;15:247. doi: 10.1186/s12870-015-0617-z (PMC4604091; doi:10.1186/s12870-015-0617-z)
Supplement: Additional file 7: — List of primers. (PDF 74 kb) [file 12870_2015_617_MOESM7_ESM.pdf]

Additional file 7: list of primers

Primers	Sequences (5‘-3‘)	Target	used for
T1A-bin2.1-F	GGTACTCCAACCCGTAAGGAAATTCG	*TaSK1*	PCR mutagenesis
T1A-bin2.1-R	CGAATTTCCTTACGGGTTGGAGTACC	*TaSK1*	PCR mutagenesis
T2A-bin2.1-F	GAACGCCAACTCGGAAGGAAATAC	*TaSK2*	PCR mutagenesis
T2A-bin2.1-R	GTATTTCCTTCCGAGTTGGCGTTC	*TaSK2*	PCR mutagenesis
BIN2-bin2.1-F	GTACACCAACTCGAAAAGAAATCCGTTGTATG	*Bin2*	PCR mutagenesis
BIN2-bin2.1-R	CATACAACGGATTTCTTTTCGAGTTGGTGTAC	*Bin2*	PCR mutagenesis
SAUR- F	GAGGAGTTTCTTGGGTGCTAAG	*SAUR-AC1*	Realtime PCR
SAUR- R2	CATAGACCGCCATGAATCCT	*SAUR-AC1*	Realtime PCR
CPD-F	CCCAAACCACTTCAAAGATGCT	*CPD*	Realtime PCR
CPD-R	GGGCCTGTCGTTACCGAGTT	*CPD*	Realtime PCR
BAS1-F	TTGGCTTCATACCGTTTGGC	*BAS1*	Realtime PCR
BAS1-R	TTACAGCGAGTGTCAATTTGGC	*BAS1*	Realtime PCR
UBQ10-F	GGCCTTGTATAATCCCTGATGAATAAG	*UBQ10*	Realtime PCR
UBQ10-R	AAAGAGATAACAGGAACGGAAACATAGT	*UBQ10*	Realtime PCR
EF1-Fnd	TGAGCACGCTCTTCTTGCTTTC	*EF-1alpha*	Realtime PCR
EF1-Rnd	GGTGGTGGCATCCATCTTGTTAC	*EF-1alpha*	Realtime PCR
SF39	GCCACAGGGATGTGAAAC	*TaSK1*	Confirmation transgene Realtime PCR
SR39	AGCTCAGGAGCACGATAG	*TaSK1*	Confirmation transgene Realtime PCR
SF86	CCGTTGCACTGCTCTTG	*TaSK2*	Confirmation transgene Realtime PCR
SR86	TTGGCCAGTTCATGCTTG	*TaSK2*	Confirmation transgene Realtime PCR
SF27	ACATATGCTCTCGCTACTATCGTGCTCCTG	*TaSK1*	RT-PCR
SR28	ACTGCCGCATTACAGTACCACTACC	*TaSK1*	RT-PCR
SF34	TTGGAAGCGCGAAAGTTCTGGTG	*TaSK2*	RT-PCR
SR34	AAACTCGGGTCCACGGCGTAAG	*TaSK2*	RT-PCR
WtubF	GCTTTCAACACCTTCTTCAGCG	Tubulin (wheat)	RT-PCR
WtubR	CCAGAGCCAGTTCCACCTCC	Tubulin (wheat)	RT-PCR
